# Pinpointing Neural Correlates of Attachment in Poly-Drug Use: A Diffusion Tensor Imaging Study

**DOI:** 10.3389/fnins.2020.00596

**Published:** 2020-06-11

**Authors:** J. Fuchshuber, H. F. Unterrainer, M. Hiebler-Ragger, K. Koschutnig, I. Papousek, E. M. Weiss, A. Fink

**Affiliations:** ^1^Center for Integrative Addiction Research (CIAR), Grüner Kreis Society, Vienna, Austria; ^2^University Clinic for Psychiatry and Psychotherapeutic Medicine, Medical University of Graz, Graz, Austria; ^3^Department of Religious Studies, University of Vienna, Vienna, Austria; ^4^Institute of Psychology, University of Graz, Graz, Austria; ^5^Institute of Psychology, University of Innsbruck, Innsbruck, Austria

**Keywords:** attachment, Diffusion Tensor Imaging, poly-drug use, regions of interest, white matter

## Abstract

An increasing amount of evidence indicates the significance of attachment in the etiology of poly-drug use disorder (PUD). The aim of this study was to investigate associations between PUD and adult attachment in particular, with a focus on white matter (WM) fiber tract integrity. For this purpose, we selected several regions-of-interest based on previous findings which were examined for their role in PUD and estimated whole-brain associations between adult attachment and WM integrity. A total sample of 144 right-handed males were investigated (Age: *M* = 27; SD = 4.66). This included a group of patients diagnosed with PUD (*n* = 70) and a group of healthy controls (HC; *n* = 74). The Adult Attachment Scales (AAS) was applied to assess attachment attitudes in participants. Diffusion Tensor Imaging was used to investigate differences in WM integrity. The findings suggest substantially less attachment security in PUD patients compared to HC. Furthermore, PUD patients exhibited reduced integrity in WM fiber tracts, most pronounced in the bilateral corticospinal tract, the fronto-occipital fasciculus, and the right inferior longitudinal fasciculus. However, these results were not controlled for comorbid depressiveness. With regard to associations between adult attachment and WM integrity, the results for PUD patients indicate a negative relationship between “Comfort with Closeness” and the structural integrity of a cluster comprising parts of the right anterior thalamic radiation, the inferior fronto-occipital fasciculus and the uncinate fasciculus. Despite being limited by the cross-sectional design of this study, the results emphasize the significance of attachment in PUD etiology, both at a behavioral and a neurological level. Largely in line with previous research, the findings revealed tentative links between adult attachment and WM fiber tracts related to cognitive and affective functions in PUD patients.

## Introduction

Attachment theory emphasizes the significance of early dyadic relationship experiences with the primary caregiver regarding the development of functional representations of the self and others (Bowlby, 1969; Mikulincer and Shaver, 2003). Based on these representations or “working models,” the individual develops secure or insecure attachment patterns which constitute rather stable traits, still traceable in adulthood (Waters et al., 2000a; Fraley, 2002). In line with Collins and Read (1990), secure adult attachment style is defined by a pattern of comfortableness with intimacy, low anxiety of being rejected and unloved, as well as the ability to depend on others and having others depending on oneself. Attachment security is associated with the ability to regulate emotions in a relatively autonomous way (Fonagy, 2018), as well as the ability to form functional and stable relationships, which allows for the regulation of emotions with the help of others (Coan, 2008). In contrast, insecure attachment patterns are linked to dysfunctional emotion regulation abilities and obstruct the formation of beneficial relationships (Schore, 2001; Belsky, 2002; Fonagy, 2010; Fuchshuber et al., 2019b). Previous research suggests, that this relationship might rely especially on the strong association between insecure attachment and increased sadness disposition (Fuchshuber et al., 2019b), as increased sadness was repeatedly observed as a typical vulnerability factor in substance use disorder (SUD; Unterrainer et al., 2017a; Fuchshuber et al., 2019a).

A multitude of empirical research has consistently identified insecure attachment as a substantial risk factor for the development of SUD (Schindler et al., 2005; Schindler and Broning, 2015). In correspondence to this, substance abuse is seen as a chemical affect regulation strategy, substituting secure attachment figures and positive inner representations, and therefore act as an artificial “secure base” for the consumer (Flores, 2004; Schindler, 2014). Initially, this has a stabilizing effect on the self and its affect regulation capabilities. Ultimately, however, substance abuse further weakens attachment and affect regulation abilities, which fosters a vicious feedback loop of increased substance abuse, gradually leading to a complete loss of control (Flores, 2004).

In line with this, insecure attachment and related problems in emotion regulation have also been found in poly-drug use disorder (PUD; Hiebler-Ragger and Unterrainer, 2019), a diagnosis highly common in individuals seeking treatment while simultaneously being associated with lower treatment success (Weigl et al., 2017).

On a neuroscientific level, the conceptualization of SUD as an attachment disorder is amplified by findings indicating striking similarities between the neuroarchitecture and neurochemistry of both social bonding and addiction in mammalians (Panksepp, 1998; Insel, 2003; Zellner et al., 2011; Burkett and Young, 2012). In correspondence to this, common neurochemical sites of action and change regarding attachment and addiction development include dopamine D1 and D2 receptors, μ-, δ-, and κ -opioid receptors and corticotropin-releasing factor (Burkett and Young, 2012). Furthermore, proposed neuroanatomic overlaps between the attachment system and areas linked to SUD etiology include the SEEKING system, which largely corresponds to the medial forebrain bundle, and the PANIC/GRIEF system, which is comprised of connections between the anterior cingulate cortex, the bed nucleus of the stria terminalis, the preoptic area, and the dorsomedial thalamus which descend to the periaqueductal gray (Coan, 2008; Panksepp, 2011; Solms et al., 2015).

Recently, neuroscientific research has increasingly focused on microstructural abnormalities in SUD patients which was investigated via diffusion tensor imaging (DTI) whereby fractional anisotropy (FA) is often utilized as a measure of structural integrity. FA is a measure of directionality of diffusion, describing the structure of myelin coating and the axonal cell membranes (Pierpaoli and Basser, 1996). A review by Arnone et al. (2006) concluded that the integrity of the corpus callosum (CC), which enables communication between cerebral hemispheres, might be particularly affected in SUDs, with most noticeable abnormalities in the genu and splenium region. However, more recent studies have found widespread reductions in FA related to opiate addiction and PUD (Bora et al., 2012; Unterrainer et al., 2016, 2017b), including the superior fasciculus longitudinalis (SLF), which connects the frontal, occipital, parietal and temporal lobes (Wang et al., 2016), and the superior corona radiata (SCR), which connects the cortex with the brain stem (Wakana et al., 2004). Moreover, Romero et al. (2010) did not find differences within the CC between patients suffering from cocaine addiction and controls, but observed lower FA in the inferior frontal white matter (WM) at the anterior-posterior commissure plane and higher FA in the anterior cingulate WM, which might indicate differences between disparate forms of addiction. Furthermore, results imply that longer duration of substance abuse is associated with lower FA (Liu et al., 2008; Bora et al., 2012). What is more, a recent study by Unterrainer et al. (2019) investigated an extensive sample of 78 PUD patients and 75 healthy controls and observed substantial impairments in the entire left and the majority of nodes of the right corticospinal tract in PUD patients. Moreover, PUD patients showed FA reductions in posterior portions of the bilateral inferior longitudinal fasciculi (ILF) and to lesser degree portions of the left thalamic radiation (TR), the right inferior fronto-occipital fasciculus (IFOF), and the right arcuate fasciculus.

With regard to the relationship between WM integrity and attachment security, the existing literature suggests that the process of increased myelination from childhood to adolescence is sensitive to the quality of parental care and early childhood attachment experiences (Daniels et al., 2013; Hanson et al., 2013; Bick et al., 2015; McCarthy-Jones et al., 2018). Considering the substantial association between early dysfunctional childhood relationships and insecure attachment in adults (Lyons-Ruth and Block, 1996; Waters et al., 2000b; Cyr et al., 2010; Fuchshuber et al., 2019b), it seems plausible that adult attachment patterns are linked to the integrity of WM fiber tracts.

In fact, several studies reported significant links between self-report measures of adult attachment and WM FA. For example, Serra et al. (2015) found positive associations between secure attachment and WM integrity in a sample of healthy male adults, including the left uncinate fasciculus (UF), left IFOF, the left SLF, and the left hippocampal region of the cingulum (HRCiC). Moreover, a study by Unterrainer et al. (2017a), which investigated a sample of male patients treated for PUD, reported tentative positive associations between the ability to depend on others and the integrity of the bilateral SCR, as well as a negative association between the anxiety of being unloved and the integrity of the right SCR. In contrast, findings by Rigon et al. (2016) indicated a positive association between attachment avoidance – which is a marker for insecure attachment – and greater structural integrity of the UF in a sample of twenty healthy female adults.

Based on the literature reviewed above, this study investigated the following hypotheses: First, PUD patients will show less secure attachment attitudes than healthy controls. Our second hypothesis is, PUD patients exhibit impaired structural integrity of white fiber tracts in several regions of interests (ROI), including the genu, splenium and body of the CC, as well as the bilateral SLF, SCR, cingulate cortex, corticospinal tract, ILF, anterior thalamic radiation (ATR), and IFOF. With regard to the role of attachment in PUD etiology, we further investigated ROIs specifically linked to attachment security in previous research adding the left UF, and the left HRCiC. Finally, we investigated possible links between WM integrity and adult attachment security. It was hypothesized that adult attachment security in healthy patients, as well as patients treated for PUD, would be correlated with increased integrity in WM fiber tracts previously associated with PUD and attachment security.

## Materials and Methods

### Participants

A total sample of 144 right-handed men between 19 and 41 years of age, composed of one clinical, and one non-clinical group, was investigated. The present study integrated samples of three different studies addressing other research questions (Unterrainer et al., 2016, 2017a; Hiebler-Ragger et al., under review). In correspondence to this, 44 participants (PUD patients: *n* = 28) were included from Unterrainer et al. (2016), 65 participants (PUD patients: *n* = 24) were included from Unterrainer et al. (2017a), and 35 (PUD patients: *n* = 17) participants were included from Hiebler-Ragger et al. (under review). The clinical group (*n* = 70) was diagnosed for PUD (F19.2) by a licensed psychiatrist according to the International Classification of Diseases version 10 (ICD 10; Dilling et al., 1991). The consumption pattern of participants of this group can be characterized by a pre-treatment chaotic use of psychoactive substances, comprising almost all substance classes (e.g., tranquilizer, opioids, stimulants, cannabis, and nicotine, etc.).

The non-clinical comparison group was comprised of students (CG; *n* = 74) who did not report any past or present psychiatric disorder or chronic disease. Psychometric assessment of the clinical subjects took place in two therapeutic facilities of the “Grüner Kreis” society, where they underwent long-term SUD treatment based on the therapeutic community concept (De Leon, 2000). The control group was behaviorally assessed at the campus of the University of Graz, Austria. All behavioral assessments were conducted via group testing. Subjects’ consent was obtained according to the Declaration of Helsinki. The study was approved by the ethics committee of the University of Graz, Austria.

### MRI Acquisition

Imaging data were acquired on a 3T Siemens Skyra (Siemens Healtheneers, Erlangen, Germany) with a 32-channel head coil. Since the investigated sample of the present study is composed of three different previous studies, two different sequence protocols were used, with slight variations in sequencing parameters. For all participants, T1-weighted images as well as diffusion-weighted images were obtained. Details of imaging parameters are shown in Table 1.

**TABLE 1:** Details of imaging parameters.

	T1		DTI	
	Study 1 and 2	Study 3	Study 1 and 2	Study 3
	44	100	44	100
TR (Repetition Time, ms)	2300	1680	8500	3036
TE (Echo Time, ms)	2.96	1.89	83	104.6
TI (Inversion Time, ms)	900	1000	–	–
FoV (Field of View, mm)	256	224	256	240
Slices (#)	176	192	64	66
Slice – Thickness (mm)	1.2	0.88	2	2.5
Gap (mm)	0.5	0.44	0	0
Matr.size	256	256	128	96
Flip angle (°)	9	8	90	86
Voxel (mm)	1 iso	0.88 iso	2 iso	2.5 iso
Directions	–	–	64	64
PAT (Parallel Acquisition Techniques)	0	0	Grappa	Multibandfactor = 3
*b*-value	–	–	1000	2000
Reverse b0	–	–	No	Yes

### Psychometric Assessment

#### Psychiatric Symptoms

The *Brief Symptom Inventory* (BSI-18; Derogatis, 2001; German version: Spitzer et al., 2011). The BSI-18 consists of 18 items assessing the amount of symptom burden in the previous seven days. The BSI-18 includes the subscales depression, anxiety and somatization. Items are rated on a 5-point Likert scale ranging from 0 “absolutely not” to 4 “very strong.” A total score “Global Severity Index” can be generated by adding the scores of every item. All scales showed good internal consistencies, with Cronbachs’s alpha ranging from 0.73 (somatization) to 0.81 (depression).

#### Adult Attachment

*The Adult Attachment Scale* (AAS; Collins and Read, 1990) is a self-report questionnaire based on the assumption that early attachment experiences form relatively stable inner attachment working models that influence individual needs and behavior in later relationships (Bowlby, 1969). The AAS consists of three subscales measuring anxiety about being rejected or unloved (“Anxiety”); comfort with closeness (“Close”); and comfort with depending on others (“Depend”). The German version of the AAS (Schmidt et al., 2004) is composed of 15 items (5 items per sub-scale) and is rated on a 5-point Likert scale ranging from 1 (“strongly disagree”) to 5 (“strongly agree”). Cronbach’s alpha for the subscales ranged between 0.90 (“Close”) and 0.72 (“Depend”).

#### Cognitive Ability

Cognitive ability was assessed by the Wonderlic Personnel Test (WPT). The WPT is a rough screening instrument for the assessment of intelligence (Wonderlic, 1999). This test requires the processing of disordered sentences, analogies, number series, word and sentence comparisons, and geometrical figures within a given time period of 12 min. The WPT contains 50 items with increasing difficulty. The total score is generated from the number of correct responses.

### Statistical Analysis

#### MRI Data Preprocessing

Data preprocessing was implemented using the software package MRtrix (Tournier et al., 2012) and FSL (Smith et al., 2004). First, data were visually inspected for artifacts and then denoised with the MRtrix command “dwidenoise” (Veraart et al., 2016). Estimation and correction of geometric distortion was carried out with FSL’s “top up” and “eddy” using the non-diffusion-weighted images (*b* value = 0) collected with reverse-phase encoding direction (Andersson and Sotiropoulos, 2016). eddy_correct was used to correct datasets with no reverse encoding direction image available. The distortion corrected diffusion-weighted images were then applied to calculate the diffusion tensors. FA and Mean Diffusivity (MD) maps were computed for each participant, using the diffusion tensor information. The FA and MD volumes of each participant were then preprocessed using the common TBSS-pipeline. Images were brought into a common space (Montreal Neurological Institute space; MN1152) via the FMRIB58_FA template using FMRIB’s non-linear registration tool (FNIRT). Then, a mean FA skeleton was created representing the centers of all tracts common to all groups. Individual FA and MD maps were then projected onto this skeleton and finally fed into voxel-wise cross-subject statistics. We applied a voxel wise permutation-based (5000 permutations) statistical approach as implemented in FSL (Smith et al., 2006). Results were corrected for multiple comparisons at *p* < 0.05 using family-wise error (FWE) correction and the threshold-free cluster enhancement (TFCE). The localization of all anatomical information is based on the “JHU ICBM-DTI-81 White-Matter Labels.”

Based on the results of previous research regarding SUD (Bora et al., 2012; Unterrainer et al., 2019), we investigated several ROI, including genu, splenium and body of the CC, as well as the bilateral SLF, SCR, cingulate cortex, corticospinal tract, ILF, ATR, IFOF, the left UF, and the left HRCiC. These ROI are based on the JHU-ICBM atlas as implemented in FSL 5. We calculated the average FA within each ROI for each subject. For the results of a whole-brain group comparison please see Unterrainer et al. (2019).

Regarding associations with adult attachment, we tested correlations between voxel-wise FA and attachment-scores, corrected for age and sequence protocols within each group.

#### Behavioral Data Analysis

For group comparisons, one-way analyses of variance were conducted. In addition, partial correlations, controlled for age and cognitive abilities, were conducted to investigate the relationships between neural and behavioral parameters. All analyses were carried out with SPSS 21. The alpha-level was set to 0.05.

## Results

### Demographics and Clinical Characteristics

As outlined in Table 2, PUD patients were older, reported fewer years of education and showed decreased cognitive abilities compared to HC participants (η*^2^* = 0.15–0.41; all *p* < 0.001). At the time of data acquisition, all PUD patients were undergoing inpatient treatment at a therapeutic community. Before taking part in the study, patients spent a mean time of 25 weeks (SD = 18.92) in their treatment facility. On average, they stated a history of drug abuse over a period of 13 years (SD = 5.79; range = 2–27; and missing values = 17). Furthermore, 30 patients were undergoing maintenance treatment (Levomethadon: *n* = 15; Morphine: *n* = 10; Buprenorphine: *n* = 1; Buprenorphine, and Naloxon: *n* = 1), while 40 patients were living in abstinence. 49 patients received psychopharmacological medication (antidepressant: *n* = 17; antipsychotic: *n* = 21; anxiolytic: *n* = 5; and other: *n* = 18).

**TABLE 2:** Group differences (ANOVA) in demographic data, cognitive ability, and psychiatric symptoms.

	CG (*n* = 74)		SUD (*n* = 70)				
Measure	*M*	SD	*M*	SD	*F*_(1, 142)_	*p*	η^2^
Age	25.24	3.38	28.90	5.09	26.09	0.000	0.16
Education (years)	13.95	2.83	11.63	2.65	25.63	0.000	0.15
WPT	28.92	6.05	18.09	6.98	99.37	0.000	0.41
**BSI-18**							
Anxiety	4.68	3.60	5.70	3.92	2.60	0.109	–
Depression	3.44	3.37	6.16	4.76	15.64	0.000	0.10
Somatization	2.23	2.79	3.61	4.02	5.74	0.018	0.04
GSI	10.36	7.74	15.47	11.22	10.14	0.002	0.07

*WPT, Wonderlic Personnel Test; BSI-18, Brief Symptom Inventory; GSI, Global Severity Index; CG, Control Group; and SUD, Patients Diagnosed with Substance Use Disorder.*

### Group Differences in Behavioral Characteristics

PUD patients reported significantly less comfort with closeness (η*^2^* = 0.17; *p* < 0.001) and less comfort with dependence on others than HC (η*^2^* = 0.17; *p* < 0.001; see Table 3). Moreover, PUD patients stated a higher anxiety of being unloved or rejected than HC (η*^2^* = 0.15; *p* < 0.001). These effect sizes regarding adult attachment security can generally be considered as large (Cohen, 1992). Moreover, PUD patients reported more depressive symptoms (η*^2^* = 0.10; *p* < 0.001), a tendency toward a higher amount of somatization symptoms (η*^2^* = 0.04; *p* < 0.02), and a significantly higher general symptom burden (η*^2^* = 0.07; *p* < 0.01).

**TABLE 3:** Group differences (ANOVA) in behavioral measures.

	CG (*n* = 74)		SUD (*n* = 70)				
Measure	*M*	SD	*M*	SD	*F*_(1, 142)_	*p*	η^2^
**AAS**							
Depend	3.64	0.62	2.99	0.81	29.24	0.000	0.17
Close	3.46	0.81	2.51	1.20	29.77	0.000	0.17
Anxiety	1.81	0.65	2.47	0.93	24.32	0.000	0.15

*AAS, Adult Attachment Scales.*

### Group Differences in Regions-of-Interests

As we found substantial group differences with regard to age and cognitive ability, estimations of group differences in WM integrity were controlled for WPT scores and age. As shown in Table 4, we found several large differences in ROIs between the investigated groups within fiber tracts of the right hemisphere. Specifically, PUD patients showed reduced WM integrity in the corticospinal tract (η*^2^* = 0.18; *p* < 0.001), the IFOF (η*^2^* = 0.17; *p* < 0.001), and the ILF (η*^2^* = 0.15; *p* < 0.001). Furthermore, we observed several ROIs in which PUD patients showed reduced FA values with medium sized differences. This included the left corticospinal tract (η*^2^* = 0.12; *p* < 0.001), the bilateral SCR (η*^2^* = 0.09–0.11; *p* < 0.01–0.001), the bilateral SLF (η*^2^* = 0.08–0.09; all *p* < 0.01), the left ILF (η*^2^* = 0.09; *p* < 0.01), the left IFOF (η*^2^* = 0.10; *p* < 0.01), and the body and genu of the CC (η*^2^* = 0.09–0.10; all *p* < 0.01).

**TABLE 4:** Group differences (ANOVA) in FA in selected ROIs, controlled for age, and cognitive ability.

	CG (*n* = 74)		SUD (*n* = 70)				
ROI	*M*	SD	*M*	SD	*F*_(1, 142)_	*p*	η^2^
Body of the CC	0.79	0.03	0.78	0.03	5.32	0.002	0.10
Genu of the CC	0.74	0.02	0.73	0.03	4.38	0.006	0.09
Splenium of the CC	0.58	0.06	0.55	0.08	2.63	0.053	–
*R* Cingulate Gyrus	0.63	0.04	0.61	0.04	2.21	0.089	–
*L* Cingulate Gyrus	0.69	0.03	0.67	0.04	3.83	0.011	–
*L* HRCiC	0.54	0.08	0.53	0.07	2.55	0.058	–
*R* Corticospinal Tract	0.64	0.02	0.62	0.02	9.99	0.000	0.18
*L* Corticospinal Tract	0.63	0.02	0.62	0.02	6.50	0.000	0.12
*R* IFOF	0.56	0.02	0.54	0.02	9.70	0.000	0.17
*L* IFOF	0.56	0.03	0.55	0.03	5.02	0.002	0.10
*R* ILF	0.54	0.02	0.52	0.03	8.46	0.000	0.15
*L* ILF	0.54	0.02	0.53	0.02	4.64	0.004	0.09
*R* SLF	0.55	0.02	0.53	0.03	4.54	0.005	0.09
*L* SLF	0.54	0.02	0.53	0.03	3.96	0.010	0.08
*R* SCR	0.54	0.02	0.52	0.02	5.55	0.001	0.11
*L* SCR	0.52	0.02	0.51	0.03	4.42	0.005	0.09
*R* ATR	0.47	0.03	0.46	0.03	1.76	0.158	–
*L* ATR	0.48	0.02	0.48	0.02	2.10	0.104	–
*L* UF	0.53	0.04	0.52	0.04	1,05	0.371	–

*Abbreviations: R, Right; L, Left; CC, Corpus Callosum; HCRiC, Hippocampal Region of Cingulum; IFOF, Inferior Fronto-Occipital Fasciculus; ILF, Inferior Longitudinal Fasciculus; SLF, Superior Longitudinal Fasciculus; SCR, Superior Corona Radiata; ATR, Anterior Thalamic Radiata; and UF, Uncinate Fasciculus.*

### Relationship Between WM Integrity and Adult Attachment in PUD Patients and HC

**FIGURE 1:**
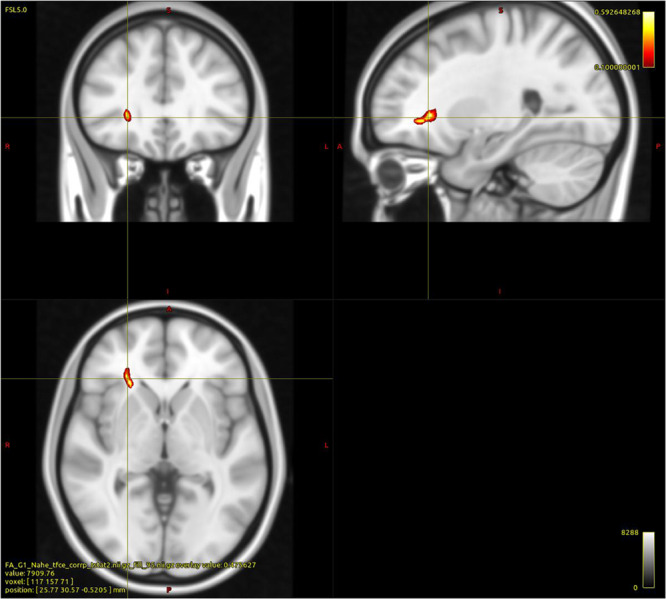
Cluster indicating a negative relationship between WM FA and “Comfort with Closeness” in PUD patients (26 Voxels; *p* < 0.05; FWE corrected; controlled for age; and study sequence protocols).

As shown in Figure 1, within the group of PUD patients the regression analysis between FA values and AAS, with controls for age, indicated a significant negative association between comfort with closeness and a cluster of 26 voxels comprising parts of the right ATR, the IFOF, and the UF (*p* < 0.05; FWE corrected). No other significant associations were observed.

## Discussion

Based on an extensive sample, this study investigated differences between healthy adults and patients suffering from PUD regarding attachment security and WM integrity, as well as associations between WM integrity and adult attachment security. Largely in accordance with our hypotheses, PUD patients showed reduced attachment security as well as reduced FA in a wide range of WM fiber tracts including: The bilateral corticospinal tract, ILF, IFOF, and to a lesser extent the bilateral SCR, SLF, and the body as well as the genu of the CC. Furthermore, the integrity of parts of the right ATR, IFOF, and UF were negatively associated with comfort with closeness in PUD patients.

In line with a significant amount of research (Schindler et al., 2005; Zellner et al., 2011; Schindler and Broning, 2015; Hiebler-Ragger et al., 2016; Unterrainer et al., 2016, 2017a) our results suggest that addiction might be understood as a dysfunctional compensation strategy against an insecure attachment organization, which utilizes psychoactive substances as soothing and seemingly “secure” attachment figures (Flores, 2004). This result corresponds to recent findings highlighting the important role of attachment in emotional functioning and affect regulation (Fuchshuber et al., 2019b; Hiebler-Ragger et al., under review), which is, in turn, frequently considered as a crucial etiological factor regarding SUD development (Cheetham et al., 2010; Khantzian, 2013). In particular, Fuchshuber et al. (2019b), was able to show that a secure attachment attitude is substantially associated with a decreased disposition toward the primary emotion SADNESS, as elaborated by Panksepp (1998). In relation to this, further research suggests that an increased disposition toward SADNESS constitutes a significant vulnerability regarding substance abuse and SUD (Zellner et al., 2011; Unterrainer et al., 2017a; Fuchshuber et al., 2019a).

On a neurological level, the present study indicates substantial and wide spread impairments regarding WM fiber tracts in PUD. The fiber tracts involved have been previously associated with affective, behavioral and motor control (Lindenberg et al., 2010; Daniels et al., 2013; Ersche et al., 2013; Jacobus et al., 2013). Among these, our results suggest that the corticospinal tract might be the most affected area. This WM tract, which connects the cortical motor areas, premotor areas and the primary somatosensory cortex to the lower motor neurons and interneurons in the spinal cord, plays a crucial role in the motoric and sensory system (Welniarz et al., 2017), and, therefore, is linked to emotional processing (LeDoux, 1993; Coombes et al., 2009; Venkatraman et al., 2017). Following on from this line of evidence, future research should aim to explore the therapeutic validity of movement training for PUD patients, as this approach is capable of facilitating the recovery of the corticospinal tract (Nakagawa et al., 2013).

Furthermore, our analysis identified substantial impairments of the IFOF and ILF in PUD patients. The former connects the occipital cortex, temporal-basal areas and superior parietal to the frontal lobe and passes the insula region (Martino et al., 2010; Wu et al., 2016). Functionally, its role has been previously associated with semantic language processing and transmission, as well as goal oriented behavior (Conner et al., 2018). The latter, which shows functional and anatomical overlaps with the IFOF (Ashtari, 2012), connects the occipital and temporal-occipital areas to the anterior temporal areas. Lesions of the ILF are associated with impairments of visual cognition (e.g., prosopagnosia, alexia, and visual agnosia), language, and emotional processing (Herbet et al., 2018). These findings are well matched with current knowledge regarding widespread neuropsychological deficits associated with SUD including impairments in verbal working memory, verbal fluency, cognitive impulsivity, decision making, visuospatial abilities, and emotional processing (van Janke Holst and Schilt, 2011; Baldacchino et al., 2012; Le Berre et al., 2017).

In correspondence to this, our results resonate with previous longitudinal studies indicating associations between intensity and duration of substance use common in PUD and abnormal WM microstructure (Lubman et al., 2007; Pfefferbaum et al., 2009; Bava et al., 2013; Qiu et al., 2013). Based on current literature, this relationship might be explained by neurotoxic effects of the consumed substances, interfering with WM development on a cellular level during adolescence, a period of life which is typically linked to the onset of problematic substance use.

With regard to links between WM fiber tracts and adult attachment, this study was not able to replicate previous findings suggesting associations between secure attachment and increased FA within the UF, SLF, and HCRiC (Serra et al., 2015) or SCR (Unterrainer et al., 2017a). Based on the present study, which employed a significantly larger sample than previous research, our results indicate that comfort with closeness – a concept which is inversely connected to avoidant attachment – is associated with reduced structural integrity of a cluster including parts of the right ATR, IFOF, and UF. This result echoes recent findings by Rigon et al. (2016) suggesting a positive correlation between avoidant attachment and UF, a WM tract which reciprocally connects the prefrontal cortex and the amygdala (Olson et al., 2013). In correspondence to this, Rigon et al. (2016) reasoned that this association might reflect increased regulation of an hyperactive amygdala, since an avoidant attachment style is characterized by a deactivating emotion regulation strategy (Mikulincer and Shaver, 2003). Furthermore, our observation supports findings by Schore (2001, 2015), who emphasizes the crucial role of the right hemisphere in the development of attachment behavior. Moreover, it resonates with the neural attachment circuits proposed by Coan (2008) and Panksepp (1998, 2011), which include connections mapping onto the fiber tracts including the ATR, IFOF, and UF. However, the question remains why this relationship was only observed within the group of PUD patients. A possible explanation for this might be that comfort with closeness in PUD patients reflects decreased caution toward others and an increased desire to fuse with others. In turn, this disinhibition might be mediated via structurally impaired fiber tracts in the observed area, which functionally compromises the communication between the prefrontal cortex and amygdala (Rigon et al., 2016). However, at this point, we cannot outright preclude the possibility, that these findings might be confounded by in-group differences within the individual substance use patterns. While all participants within the PUD group showed a similarly chaotic pattern of substance use previous to treatment, including the chronic consumption of a wide range of substance, future studies need to asses the distribution of used substances in more detail. Therefore, further research well have to test this preliminary interpretation.

Regarding further limitations of this study, it is important to note that our cross-sectional design limits the causal interpretation of the relationship between WM integrity and PUD. Therefore, future research should aim at implementing longitudinal studies. Furthermore, this study solely included right handed male participants, which reduces the generalizability of these results with regard to the general population. What is more, our clinical sample exclusively investigated patients diagnosed for PUD, hence these results should not be generalized for other SUD patients. In addition, this study employed a self-rated measure of adult attachment. As these measurements might be prone to self-perception distortions and only capture consciously available attachment attitudes, future studies might utilize semi structured interviews (e.g., the Adult Attachment Interview; George et al., 1996) to assess adult attachment in more detail. On a similar note, participants within the PUD group showed significantly more depressive symptoms and are very likely to show a high amount of comorbid symptoms (Grant et al., 2016). With this in mind, future studies need to investigate the psychiatric symptom burden in a more detailed manner. Therefore, more extensive and objective diagnostic tools [e.g., the SCID-I and II; Lobbestael et al. (2011)] will be implemented in future studies. Moreover, with regard to previous research (Wurmser, 1978; Zellner et al., 2011; Khantzian, 2013; Solms et al., 2015), which emphasizes depression as a disorder which commonly underlies SUDs and is fended of by chronic substance consumption, the results were not controlled for depressiveness.

Overall, the present study provides an important step in the mapping of the neurological correlates of adult attachment in PUD patients. Therein, the results emphasize the importance of attachment in PUD etiology. While this suggests the need to integrate attachment oriented approaches in PUD therapeutic strategies, longitudinal studies focused on attachment changes, and neuroplasticity in this context are still very much needed.

## Data Availability Statement

The raw data supporting the conclusions of this article will be made available by the authors, without undue reservation, to any qualified researcher.

## Ethics Statement

This study was carried out in accordance with the recommendations of the ethics guidelines of the University of Graz. The protocol was approved by the Ethics Committee of the University of Graz. All subjects gave written informed consent in accordance with the Declaration of Helsinki.

## Author Contributions

JF, HU, KK, and AF conceptualized the study. MH-R, JF, KK, HU, and AF collected, analyzed, and interpreted the data. JF and HU drafted the manuscript. IP, EW, and AF critically reviewed the manuscript. All authors gave their final approval of the manuscript.

## Conflict of Interest

The authors declare that the research was conducted in the absence of any commercial or financial relationships that could be construed as a potential conflict of interest.

## References

[B1] Andersson J. L. R. Sotiropoulos S. N. (2016). An integrated approach to correction for off-resonance effects and subject movement in diffusion MR imaging. *Neuroimage* 125 1063–1078. 10.1016/j.neuroimage.2015.10.019 26481672 PMC4692656

[B2] Arnone D. Abou-Saleh M. T. Barrick T. R. (2006). Diffusion tensor imaging of the corpus callosum in addiction. *Neuropsychobiology* 54 107–113. 10.1159/000096992 17108711

[B3] Ashtari M. (2012). Anatomy and functional role of the inferior longitudinal fasciculus: a search that has just begun. *Dev. Med. Child Neurol.* 54 6–7. 10.1111/j.1469-8749.2011.04122.x 22098073

[B4] Baldacchino A. Balfour D. J. K. Passetti F. Humphris G. Matthews K. (2012). Neuropsychological consequences of chronic opioid use: a quantitative review and meta-analysis. *Neurosci. Biobehav. Rev.* 36 2056–2068. 10.1016/j.neubiorev.2012.06.006 22771335

[B5] Bava S. Jacobus J. Thayer R. E. Tapert S. F. (2013). Longitudinal changes in white matter integrity among adolescent substance users. *Alcohol. Clin. Exp. Res.* 37 E181–E189. 23240741 10.1111/j.1530-0277.2012.01920.x PMC3548057

[B6] Belsky J. (2002). Developmental origins of attachment styles. *Attach. Hum. Dev.* 4 166–170. 10.1080/14616730210157510 12467508

[B7] Bick J. Zhu T. Stamoulis C. Fox N. A. Zeanah C. Nelson C. A. (2015). Effect of early institutionalization and foster care on long-term white matter development: a randomized clinical trial. *JAMA Pediatr.* 169 211–219. 25622303 10.1001/jamapediatrics.2014.3212 PMC4413892

[B8] Bora E. Yücel M. Fornito A. Pantelis C. Harrison B. J. Cocchi L. (2012). White matter microstructure in opiate addiction. *Addict. Biol.* 17 141–148. 10.1111/j.1369-1600.2010.00266.x 21070508

[B9] Bowlby J. (1969). *Attachment and Loss: Attachment.* New York, NY: Basic Books.

[B10] Burkett J. P. Young L. J. (2012). The behavioral, anatomical and pharmacological parallels between social attachment, love and addiction. *Psychopharmacology* 224 1–26. 10.1007/s00213-012-2794-x 22885871 PMC3469771

[B11] Cheetham A. Allen N. B. Yücel M. Lubman D. I. (2010). The role of affective dysregulation in drug addiction. *Clin. Psychol. Rev.* 30 621–634. 10.1016/j.cpr.2010.04.005 20546986

[B12] Coan J. A. (2008). Toward a neuroscience of attachment. *Handb. Attach.* 2 241–265.

[B13] Cohen J. (1992). A power primer. *Psychol. Bull.* 112 155–159. 19565683 10.1037//0033-2909.112.1.155

[B14] Collins N. L. Read S. J. (1990). Adult attachment, working models, and relationship quality in dating couples. *J. Pers. Soc. Psychol.* 58 644–663. 10.1037/0022-3514.58.4.644 14570079

[B15] Conner A. K. Briggs R. G. Sali G. Rahimi M. Baker C. M. Burks J. D. (2018). A connectomic atlas of the human cerebrum—chapter 13: tractographic description of the inferior fronto-occipital fasciculus. *Oper. Neurosurg.* 15 S436–S443. 10.1093/ons/opy267 PMC6890527 30260438

[B16] Coombes S. A. Tandonnet C. Fujiyama H. Janelle C. M. Cauraugh J. H. Summers J. J. (2009). Emotion and motor preparation: a transcranial magnetic stimulation study of corticospinal motor tract excitability. *Cogn. Affect. Behav. Neurosci.* 9 380–388. 10.3758/cabn.9.4.380 19897791

[B17] Cyr C. Euser E. M. Bakermans-Kranenburg M. J. van Ijzendoorn M. H. (2010). Attachment security and disorganization in maltreating and high-risk families: a series of meta-analyses. *Dev. Psychopathol.* 22 87–108. 10.1017/s0954579409990289 20102649

[B18] Daniels J. K. Lamke J. -P. Gaebler M. Walter H. Scheel M. (2013). White matter integrity and its relationship to PTSD and childhood trauma—a systematic review and meta-analysis. *ıDepress. Anxiety* 30 207–216. 10.1002/da.22044 23319445

[B19] De Leon G. (2000). *The Therapeutic Community: Theory, Model, And Method.* New York, NY: Springer Publishing Co.

[B20] Derogatis L. R. (2001). *Brief Symptom Inventory 18.* Baltimore: Johns Hopkins University.

[B21] Dilling H. Mombour W. Schmidt M. H. (1991). *Internationale Klassifikation Psychischer Störungen : ICD-10, Kapitel V (F), Klinisch-Diagnostische Leitlinien/hrsg.* Bern: Huber Hans Available online at: https://apps.who.int/iris/handle/10665/38221

[B22] Ersche K. D. Williams G. B. Robbins T. W. Bullmore E. T. (2013). Meta-analysis of structural brain abnormalities associated with stimulant drug dependence and neuroimaging of addiction vulnerability and resilience. *Curr. Opin. Neurobiol.* 23 615–624. 10.1016/j.conb.2013.02.017 23523373

[B23] Flores P. J. (2004). *Addiction As An Attachment Disorder.* Lanham: Jason Aronson.

[B24] Fonagy P. (2010). Psychotherapy research: do we know what works for whom? *Br. J. Psychiatry.* 197 83–85. 10.1192/bjp.bp.110.079657 20679254

[B25] Fonagy P. (2018). *Affect Regulation, Mentalization And The Development Of The Self.* Abingdon: Routledge.

[B26] Fraley C. (2002). Attachment stability from infancy to adulthood. Meta-analysis and dynamic modeling of developmental mechanisms. *Pers. Soc. Psychol. Rev.* 6 123–151. 10.1207/s15327957pspr0602_03 26627889

[B27] Fuchshuber J. Hiebler-Ragger M. Kresse A. Kapfhammer H. P. Unterrainer H. F. (2019a). Do primary emotions predict psychopathological symptoms? A multi-group path analysis. *Front. Psychiatry* 2019:610. 10.3389/fpsyt.2019.00610 31543836 PMC6730598

[B28] Fuchshuber J. Hiebler-Ragger M. Kresse A. Kapfhammer H. P. Unterrainer H. F. (2019b). The influence of attachment styles and personality organization on emotional functioning after childhood trauma. *Front. Psychiatry* 2019:643. 10.3389/fpsyt.2019.00643 31543844 PMC6739441

[B29] George C. Kaplan N. Main M. (1996). *Adult Attachment Interview.* Berkeley: University of California.

[B30] Grant B. F. Saha T. D. Ruan W. J. Goldstein R. B. Chou S. P. Jung J. (2016). Epidemiology of DSM-5 drug use disorder. Results from the national epidemiologic survey on alcohol and related conditions–III. *JAMA Psychiatry* 73 39–47. 26580136 10.1001/jamapsychiatry.2015.2132 PMC5062605

[B31] Hanson J. L. Adluru N. Chung M. K. Alexander A. L. Davidson R. J. Pollak S. D. (2013). Early neglect is associated with alterations in white matter integrity and cognitive functioning. *Child. Dev.* 84 1566–1578. 10.1111/cdev.12069 23480812 PMC3690164

[B32] Herbet G. Zemmoura I. Duffau H. (2018). Functional anatomy of the inferior longitudinal fasciculus: from historical reports to current hypotheses. *Front. Neuroanat.* 12:77. 10.3389/fnana.2018.00077 30283306 PMC6156142

[B33] Hiebler-Ragger M. Unterrainer H.-F. (2019). The role of attachment in poly-drug use disorder: an overview of the literature, recent findings and clinical implications. *Front. Psychiatry* 10:579. 10.3389/fpsyt.2019.00579 31507461 PMC6720034

[B34] Hiebler-Ragger M. Unterrainer H.-F. Rinner A. Kapfhammer H.-P. (2016). Insecure attachment styles and increased borderline personality organization in substance use disorders. *Psychopathology* 49 341–344. 10.1159/000448177 27631792

[B35] Insel T. R. (2003). Is social attachment an addictive disorder? *Physiol. Behav.* 79 351–357. 10.1016/s0031-9384(03)00148-3 12954430

[B36] Jacobus J. Thayer R. E. Trim R. S. Bava S. Frank L. R. Tapert S. F. (2013). White matter integrity, substance use, and risk taking in adolescence. *ıPsychol. Addict. Behav.* 27 431–442. 10.1037/a0028235 22564204 PMC3416962

[B37] Khantzian E. J. (2013). Addiction as a self-regulation disorder and the role of self-medication. *Addiction* 108 668–669. 10.1111/add.12004 23496062

[B38] Le Berre A.-P. Fama R. Sullivan E. V. (2017). Executive functions, memory, and social cognitive deficits and recovery in chronic alcoholism: a critical review to inform future research. *Alcohol. Clin. Exp. Res.* 41 1432–1443. 10.1111/acer.13431 28618018 PMC5531758

[B39] LeDoux J. E. (1993). Emotional memory systems in the brain. *Behav. Brain Res.* 58 69–79. 10.1016/0166-4328(93)90091-4 8136051

[B40] Lindenberg R. Renga V. Zhu L. L. Betzler F. Alsop D. Schlaug G. (2010). Structural integrity of corticospinal motor fibers predicts motor impairment in chronic stroke. *Neurology* 74 280–287. 10.1212/wnl.0b013e3181ccc6d9 20101033 PMC3122304

[B41] Liu H. Li L. Hao Y. Cao D. Xu L. Rohrbaugh R. (2008). Disrupted white matter integrity in heroin dependence. A controlled study utilizing diffusion tensor imaging. *Am. J. Drug Alcohol. Abuse* 34 562–575. 10.1080/00952990802295238 18720268

[B42] Lobbestael J. Leurgans M. Arntz A. (2011). Inter-rater reliability of the structured clinical interview for DSM-IV axis I disorders (SCID I) and axis II DISORDERS (SCID II). *Clin. Psychol. Psychother.* 18 75–79. 10.1002/cpp.693 20309842

[B43] Lubman D. I. Yücel M. Hall W. D. (2007). Substance use and the adolescent brain: a toxic combination? *J. Psychopharmacol.* 21 792–794. 10.1177/0269881107078309 17984159

[B44] Lyons-Ruth K. Block D. (1996). The disturbed caregiving system: relations among childhood trauma, maternal caregiving, and infant affect and attachment. *Infant Ment. Health J.* 17 257–275. 10.1002/(sici)1097-0355(199623)17:3<257::aid-imhj5>3.0.co;2-l

[B45] Martino J. Brogna C. Robles S. G. Vergani F. Duffau H. (2010). Anatomic dissection of the inferior fronto-occipital fasciculus revisited in the lights of brain stimulation data. *Cortex* 46 691–699. 10.1016/j.cortex.2009.07.015 19775684

[B46] McCarthy-Jones S. Oestreich L. K. L. Lyall A. E. Kikinis Z. Newell D. T. Savadjiev P. (2018). Childhood adversity associated with white matter alteration in the corpus callosum, corona radiata, and uncinate fasciculus of psychiatrically healthy adults. *Brain Imaging Behav.* 12 449–458. 10.1007/s11682-017-9703-1 28341872 PMC6310006

[B47] Mikulincer M. Shaver P. R. (2003). The attachment behavioral system in adulthood: activation, psychodynamics, and interpersonal processes. *Adv. Exp. Soc. Psychol.* 35 56–152.

[B48] Nakagawa H. Ueno M. Itokazu T. Yamashita T. (2013). Bilateral movement training promotes axonal remodeling of the corticospinal tract and recovery of motor function following traumatic brain injury in mice. *Cell Death Dis.* 4:e534. 10.1038/cddis.2013.62 23470541 PMC3613840

[B49] Olson I. R. McCoy D. Klobusicky E. Ross L. A. (2013). Social cognition and the anterior temporal lobes: a review and theoretical framework. ı*Soc. Cogn. Affect. Neurosci.* 8 123–133. 10.1093/scan/nss119 23051902 PMC3575728

[B50] Panksepp J. (1998). *Affective Neuroscience: The Foundations Of Human And Animal Emotions.* Oxford: Oxford university press.

[B51] Panksepp J. (2011). The basic emotional circuits of mammalian brains: do animals have affective lives? *Neurosci. Biobehav. Rev.* 35 1791–1804. 10.1016/j.neubiorev.2011.08.003 21872619

[B52] Pfefferbaum A. Rosenbloom M. Rohlfing T. Sullivan E. V. (2009). Degradation of association and projection white matter systems in alcoholism detected with quantitative fiber tracking. *Biol. Psychiatry* 65 680–690. 10.1016/j.biopsych.2008.10.039 19103436 PMC2663629

[B53] Pierpaoli C. Basser P. J. (1996). Toward a quantitative assessment of diffusion anisotropy. *Magn. Reson. Med.* 36 893–906. 10.1002/mrm.1910360612 8946355

[B54] Qiu Y. Jiang G. Su H. Lv X. Zhang X. Tian J. (2013). Progressive white matter microstructure damage in male chronic heroin dependent individuals: a DTI and TBSS study. *PLoS One* 8:e63212. 10.1371/journal.pone.0063212 23650554 PMC3641135

[B55] Rigon A. Duff M. C. Voss M. W. (2016). Structural and functional neural correlates of self-reported attachment in healthy adults: evidence for an amygdalar involvement. *Brain Imaging Behav.* 10 941–952. 10.1007/s11682-015-9446-9 26334650

[B56] Romero M. J. Asensio S. Palau C. Sanchez A. Romero F. J. (2010). Cocaine addiction. Diffusion tensor imaging study of the inferior frontal and anterior cingulate white matter. *Psychiatry Res. Neuroimag.* 181 57–63. 10.1016/j.pscychresns.2009.07.004 19959341

[B57] Schindler A. (2014). “Bindung und Sucht–theoretische modelle, empirische zusammenhänge und therapeutische implikationen,” in *Bindung und Sucht*, ed. Brisch K. H. (Stuttgart: Klett-Cotta).

[B58] Schindler A. Broning S. (2015). A review on attachment and adolescent substance abuse. empirical evidence and implications for prevention and treatment. *Subst. Abus.* 36 304–313. 10.1080/08897077.2014.983586 25424652

[B59] Schindler A. Thomasius R. Sack P.-M. Gemeinhardt B. KÜStner U. Eckert J. (2005). Attachment and substance use disorders: a review of the literature and a study in drug dependent adolescents. *Attach. Hum. Dev.* 7 207–228. 10.1080/14616730500173918 16210236

[B60] Schmidt S. Strauss B. Höger D. Brähler E. (2004). The adult attachment scale (AAS)-psychometric evaluation and normation of the german version. *Psychother. Psychosom. Med. Psychol.* 54 375–382. 10.1055/s-2003-815000 15343479

[B61] Schore A. N. (2001). The effects of early relational trauma on right brain development, affect regulation, and infant mental health. *Infant. Ment. Health J.* 22 201–269. 10.1002/1097-0355(200101/04)22:1<201::aid-imhj8>3.0.co;2-9

[B62] Schore A. N. (2015). *Affect Regulation And The Origin Of The Self: The Neurobiology Of Emotional Development.* Abingdon: Routledge.

[B63] Serra M. Pisapia N. de Rigo P. Papinutto N. Jager J. Bornstein M. H. (2015). Secure attachment status is associated with white matter integrity in healthy young adults. *Neuroreport* 26:1106. 10.1097/wnr.0000000000000479 26559724 PMC4646732

[B64] Smith S. M. Jenkinson M. Johansen-Berg H. Rueckert D. Nichols T. E. Mackay C. E. (2006). Tract-based spatial statistics: voxelwise analysis of multi-subject diffusion data. *Neuroimage* 31 1487–1505. 10.1016/j.neuroimage.2006.02.024 16624579

[B65] Smith S. M. Jenkinson M. Woolrich M. W. Beckmann C. F. Behrens T. E. J. Johansen-Berg H. (2004). Advances in functional and structural MR image analysis and implementation as FSL. *Neuroimage* 23 208–219. 10.1016/j.neuroimage.2004.07.051 15501092

[B66] Solms M. Pantelis E. Panksepp J. (2015). “Neuropsychoanalytic notes on addiction,” in *The Feeling Brain: Selected Papers On Neuropsychoanalysis*, ed. Solms M. (London: Karnac Books), 109–119. 10.4324/9780429481758-8

[B67] Spitzer C. Hammer S. Löwe B. Grabe H. J. Barnow S. Rose M. (2011). Die kurzform des brief symptom inventory (BSI-18). Erste befunde zu den psychometrischen kennwerten der deutschen version. *Fortschr. Neurol. Psychiatr.* 79 517–523. 10.1055/s-0031-1281602 21870312

[B68] Tournier J.-D. Calamante F. Connelly A. (2012). MRtrix: diffusion tractography in crossing fiber regions. *Int. J. Imag. Syst. Tech.* 22 53–66. 10.1002/ima.22005

[B69] Unterrainer H. F. Hiebler M. Ragger K. Froehlich L. Koschutnig K. Schoeggl H. (2016). White matter integrity in polydrug users in relation to attachment and personality. A controlled diffusion tensor imaging study. *Brain Imaging Behav.* 10 1096–1107. 10.1007/s11682-015-9475-4 26542619

[B70] Unterrainer H. F. Hiebler-Ragger M. Koschutnig K. Fuchshuber J. Ragger K. Perchtold C. (2019). Brain structure alterations in poly-drug use. reduced cortical thickness and white matter impairments in regions associated with affective, cognitive and motor functions. *Front. Psychiatry* 10:667. 10.3389/fpsyt.2019.00667 31616326 PMC6763614

[B71] Unterrainer H. F. Hiebler-Ragger M. Koschutnig K. Fuchshuber J. Tscheschner S. Url M. (2017a). Addiction as an attachment disorder. white matter impairment is linked to increased negative affective states in poly-drug use. *Front. Hum. Neurosci.* 11:208. 10.3389/fnhum.2017.00208 28503141 PMC5408064

[B72] Unterrainer H. F. Hiebler-Ragger M. Rogen L. Kapfhammer H. P. (2017b). *Sucht Als Bindungsstörung.* Berlin: Springer. 10.1007/s00115-017-0462-4 29209752

[B73] van Janke Holst R. Schilt T. (2011). Drug-related decrease in neuropsychological functions of abstinent drug users. *Curr. Drug Abuse Rev.* 4 42–56. 10.2174/1874473711104010042 21466500

[B74] Venkatraman A. Edlow B. L. Immordino-Yang M. H. (2017). The brainstem in emotion: a review. *Front. Neuroanat.* 11:15 10.3389/fnhum.2017.0015 PMC5343067 28337130

[B75] Veraart J. Novikov D. S. Christiaens D. Ades-Aron B. Sijbers J. Fieremans E. (2016). Denoising of diffusion MRI using random matrix theory. *Neuroimage* 142 394–406. 10.1016/j.neuroimage.2016.08.016 27523449 PMC5159209

[B76] Wakana S. Jiang H. Nagae-Poetscher L. M. van Zijl P. C. M. Mori S. (2004). Fiber tract–based atlas of human white matter anatomy. *Radiology* 230 77–87. 10.1148/radiol.2301021640 14645885

[B77] Wang X. Pathak S. Stefaneanu L. Yeh F.-C. Li S. Fernandez-Miranda J. C. (2016). Subcomponents and connectivity of the superior longitudinal fasciculus in the human brain. *Brain Struct. Funct.* 221 2075–2092. 10.1007/s00429-015-1028-5 25782434

[B78] Waters E. Hamilton C. E. Weinfield N. S. (2000a). The stability of attachment security from infancy to adolescence and early adulthood: general introduction. *Child. Dev.* 71 678–683. 10.1111/1467-8624.00175 10953933

[B79] Waters E. Merrick S. Treboux D. Crowell J. Albersheim L. (2000b). Attachment security in infancy and early adulthood: a twenty-year longitudinal study. *Child. Dev.* 71 684–689. 10.1111/1467-8624.00176 10953934

[B80] Weigl M. Anzenberger J. Grabenhofer-Eggerth A. Horvath I. Schmutterer I. Strizek J. (2017). *Bericht Zur Drogensituation 2017.* Vienna: Gesundheit Österreich.

[B81] Welniarz Q. Dusart I. Roze E. (2017). The corticospinal tract: Evolution, development, and human disorders. *Dev. Neurobiol.* 77 810–829. 10.1002/dneu.22455 27706924

[B82] Wonderlic E. (1999). *Wonderlic Personnel Test and Scholastic Level Exam, User’s Manual.* Libertyville: Wonderlic Test.

[B83] Wu Y. Sun D. Wang Y. Wang Y. (2016). Subcomponents and connectivity of the inferior fronto-occipital fasciculus revealed by diffusion spectrum imaging fiber tracking. *Front. Neuroanat.* 10:88 10.3389/fnhum.2017.0088 PMC5033953 27721745

[B84] Wurmser L. (1978). *The Hidden Dimension: Psychodynamics in Compulsive Drug Use.* Lanham: J. Aronson.

[B85] Zellner M. R. Watt D. F. Solms M. Panksepp J. (2011). Affective neuroscientific and neuropsychoanalytic approaches to two intractable psychiatric problems. why depression feels so bad and what addicts really want. *Neurosci. Biobehav. Rev.* 35 2000–2008. 10.1016/j.neubiorev.2011.01.003 21241736

